# *catena*-Poly[[[(1,10-phenanthroline-κ^2^ *N*,*N*′)zinc]-μ-pyridine-2,3-dicarboxyl­ato-κ^4^ *N*,*O* ^2^:*O* ^2′^,*O* ^3^] monohydrate]

**DOI:** 10.1107/S1600536811020344

**Published:** 2011-06-04

**Authors:** Zhen-Hua Qin, Shu-Fang Lou, Na Wang, Fu-Yu Wang

**Affiliations:** a Shangqiu Medical College, Shangqiu, Henan 476100, People’s Republic of China

## Abstract

In the title complex, {[Zn(C_7_H_3_NO_4_)(C_12_H_8_N_2_)]·H_2_O}_*n*_, the Zn^II^ ion is in a distorted octa­hedral environment, defined by two N atoms from a chelating 1,10-phenanthroline (phen) ligand and one N atom and three O atoms from two pyridine-2,3-dicarboxyl­ate (2,3-pydc) ligands. The bridging 2,3-pydc ligands connect the Zn^II^ ions into a chain extending along [010]. O—H⋯O hydrogen bonds between the uncoordinated water mol­ecules and the uncoordinated carboxyl­ate O atoms, as well as π–π inter­actions between the pyridine rings of the phen ligands [centroid–centroid distance = 3.557 (2) Å], are observed.

## Related literature

For complexes based on pyridine-2,3-dicarb­oxy­lic acid, see: Du *et al.* (2008 ▶); Han *et al.* (2006 ▶); Li & Li (2004 ▶); Patrick *et al.* (2003 ▶). For a description of the Cambridge Structural Database, see: Allen (2002 ▶). For a related structure, see: Shit *et al.* (2008 ▶).
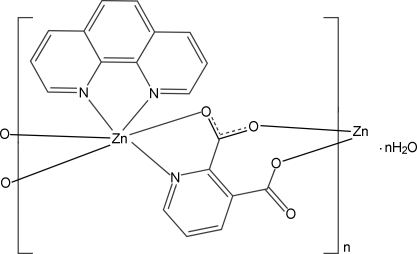


## Experimental

### 

#### Crystal data

[Zn(C_7_H_3_NO_4_)(C_12_H_8_N_2_)]·H_2_O
*M* *_r_* = 428.69
Orthorhombic, 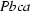

*a* = 12.2099 (8) Å
*b* = 11.6922 (8) Å
*c* = 23.2513 (15) Å
*V* = 3319.4 (4) Å^3^
*Z* = 8
Mo *K*α radiation
μ = 1.52 mm^−1^
*T* = 293 K
0.23 × 0.19 × 0.12 mm

#### Data collection

Bruker APEXII CCD diffractometer
Absorption correction: multi-scan (*SADABS*; Sheldrick, 1996 ▶) *T*_min_ = 0.721, *T*_max_ = 0.839
30212 measured reflections
3802 independent reflections
3010 reflections with *I* > 2σ(*I*)
*R*_int_ = 0.040

#### Refinement

*R*[*F* ^2^ > 2σ(*F* ^2^)] = 0.051
*wR*(*F* ^2^) = 0.175
*S* = 1.06
3802 reflections
253 parameters
H-atom parameters constrained
Δρ_max_ = 0.76 e Å^−3^
Δρ_min_ = −1.02 e Å^−3^

### 

Data collection: *APEX2* (Bruker, 2007 ▶); cell refinement: *SAINT* (Bruker, 2007 ▶); data reduction: *SAINT*; program(s) used to solve structure: *SHELXS97* (Sheldrick, 2008 ▶); program(s) used to refine structure: *SHELXL97* (Sheldrick, 2008 ▶); molecular graphics: *SHELXTL* (Sheldrick, 2008 ▶); software used to prepare material for publication: *SHELXTL*.

## Supplementary Material

Crystal structure: contains datablock(s) I, global. DOI: 10.1107/S1600536811020344/hy2421sup1.cif

Structure factors: contains datablock(s) I. DOI: 10.1107/S1600536811020344/hy2421Isup2.hkl

Additional supplementary materials:  crystallographic information; 3D view; checkCIF report

## Figures and Tables

**Table 1:** Selected bond lengths (Å)

Zn1—O1	2.102 (2)
Zn1—O2^i^	2.110 (2)
Zn1—O4^i^	2.062 (2)
Zn1—N1	2.142 (3)
Zn1—N2	2.139 (3)
Zn1—N3	2.131 (3)

Symmetry code: (i) 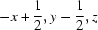.

**Table 2:** Hydrogen-bond geometry (Å, °)

*D*—H⋯*A*	*D*—H	H⋯*A*	*D*⋯*A*	*D*—H⋯*A*
O1*W*—H1*WA*⋯O3	0.85	2.22	3.022 (5)	157
O1*W*—H1*WB*⋯O3^ii^	0.85	2.42	3.215 (5)	157

Symmetry code: (ii) 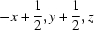.

**Fig. 1.**
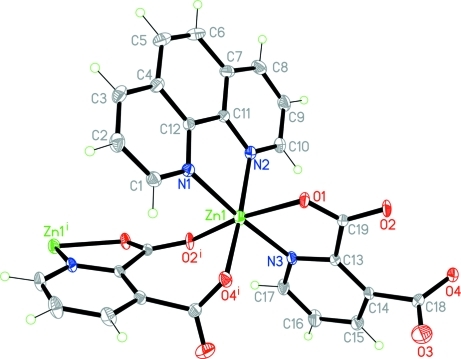
Part of the chain structure in the title complex. Displacement ellipsoids are drawn at the 30% probability level. Uncoordinated water molecule has been omitted. [Symmetry code: (i) 1/2 - x, -1/2 + y, z.]

**Fig. 2.**
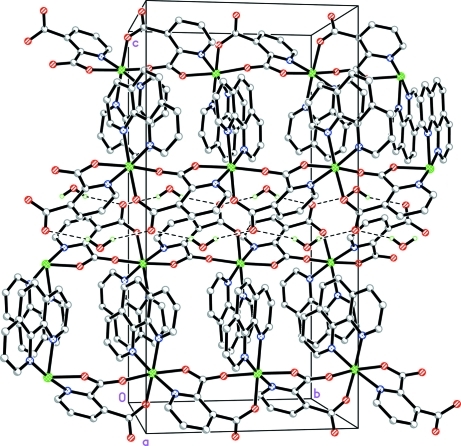
The packing diagram of the title complex. Dashed lines denote hydrogen bonds. H atoms not involving in hydrogen bonds have been omitted.

## supplementary crystallographic information

### Comment

Pyridine-carboxylic acids are widely used to form supramolecular architectures due to their diverse coordination modes such as monodentate terminal, monodentate bridging and bidentate chelating. Recently, pyridine-2,3-dicarboxylic acid (2,3-H_2_pydc) acts as a bidentate and bridging ligand to construct one-dimensional chain, two-dimensional layer and three-dimensional structures (Du *et al.*, 2008; Han *et al.*, 2006; Li & Li, 2004; Patrick *et al.*, 2003). Herein, a one-dimensional polymeric complex based on 2,3-pydc was investigated.

The title compound is composed of polymeric chains and uncoordinated water molecules. The asymmetric unit contains one Zn^II^ ion, one uncoordinated water molecule, one 2,3-pydc ligand and one 1,10-phenanthroline (1,10-phen) ligand. The coordination geometry of the Zn^II^ ion can be described as distorted octahedral, which is defined by three N and three O atoms from one 1,10-phen and two 2,3-pydc ligands, with Zn—N and Zn—O distances of 2.131 (3)–2.142 (3) and 2.062 (2)–2.110 (2) Å, respectively (Fig. 1, Table 1). As shown in Fig. 2, adjacent Zn^II^ ions are bridged by 2,3-pydc ligands, which adopt a *syn–anti* configuration, with an internuclear Zn···Zn distance of 5.9206 (6) Å, building an infinite one-dimensional architecture. The 1,10-phen molecule serves as a bidentate chelating ligand to coordinate to the Zn^II^ ion. In the chain, the dihedral angle between the adjacent 1,10-phen ligands is 65.24 (3)°.

According to the search results in CSD database (Conquest version 1.12) (Allen, 2002), an isomorphous complex {[Mn(C_7_H_3_NO_4_)(C_12_H_8_N_2_)].H_2_O}_n_ is reported (Shit *et al.*, 2008).

### Experimental

A mixture of pyridine-2,3-dicarboxylic acid (0.2 mmol), Zn(CH_3_COOH)_2_.2H_2_O (0.2 mmol), 1,10-phenanthroline (0.1 mmol) and 10 ml me thanol/distilled water(*v*/*v* 9:2) sealed in a 25 ml Teflon-lined stainless steel autoclave was kept at 393 K for three days and then cooled to room temperature. Colorless crystals suitable for X-ray analysis were obtained.

### Refinement

H atoms of aromatic rings were positioned geometrically and refined as riding atoms, with C—H = 0.93 Å and *U*_iso_(H) = 1.2*U*_eq_(C). H atoms of water molecule were found in a difference Fourier map and refined as riding atoms, with O—H = 0.85 Å and *U*_iso_(H) = 1.5*U*_eq_(O).

### Crystal data

**Table:**

[Zn(C_7_H_3_NO_4_)(C_12_H_8_N_2_)]·H_2_O	*F*(000) = 1744
*M**_r_* = 428.69	*D*_x_ = 1.716 Mg m^−^^3^
Orthorhombic, *P**b**c**a*	Mo *K*α radiation, λ = 0.71073 Å
Hall symbol: -P 2ac 2ab	Cell parameters from 4015 reflections
*a* = 12.2099 (8) Å	θ = 1.8–28.3°
*b* = 11.6922 (8) Å	µ = 1.52 mm^−^^1^
*c* = 23.2513 (15) Å	*T* = 293 K
*V* = 3319.4 (4) Å^3^	Block, colorless
*Z* = 8	0.23 × 0.19 × 0.12 mm

### Data collection

**Table:**

Bruker APEXII CCD diffractometer	3802 independent reflections
Radiation source: fine-focus sealed tube	3010 reflections with *I* > 2σ(*I*)
graphite	*R*_int_ = 0.040
φ and ω scans	θ_max_ = 27.5°, θ_min_ = 1.8°
Absorption correction: multi-scan (*SADABS*; Sheldrick, 1996)	*h* = −15→15
*T*_min_ = 0.721, *T*_max_ = 0.839	*k* = −15→15
30212 measured reflections	*l* = −29→29

### Refinement

**Table:**

Refinement on *F*^2^	Primary atom site location: structure-invariant direct methods
Least-squares matrix: full	Secondary atom site location: difference Fourier map
*R*[*F*^2^ > 2σ(*F*^2^)] = 0.051	Hydrogen site location: inferred from neighbouring sites
*wR*(*F*^2^) = 0.175	H-atom parameters constrained
*S* = 1.06	*w* = 1/[σ^2^(*F*_o_^2^) + (0.1103*P*)^2^ + 4.4449*P*] where *P* = (*F*_o_^2^ + 2*F*_c_^2^)/3
3802 reflections	(Δ/σ)_max_ = 0.001
253 parameters	Δρ_max_ = 0.76 e Å^−^^3^
0 restraints	Δρ_min_ = −1.02 e Å^−^^3^

### Fractional atomic coordinates and isotropic or equivalent isotropic displacement parameters (Å^2^)

**Table:**

	*x*	*y*	*z*	*U*_iso_*/*U*_eq_	
Zn1	0.21166 (4)	0.05837 (3)	0.378838 (18)	0.02796 (19)	
O1	0.1506 (2)	0.22624 (19)	0.37472 (10)	0.0250 (5)	
O2	0.19575 (19)	0.4075 (2)	0.38954 (11)	0.0248 (5)	
O3	0.2708 (3)	0.4531 (3)	0.53194 (15)	0.0586 (10)	
O4	0.3580 (2)	0.53247 (19)	0.45856 (10)	0.0288 (5)	
N1	0.0847 (2)	−0.0270 (2)	0.33197 (12)	0.0230 (6)	
N2	0.2625 (2)	0.0835 (2)	0.29172 (12)	0.0242 (6)	
N3	0.3382 (2)	0.1532 (2)	0.42019 (11)	0.0217 (5)	
C1	−0.0016 (3)	−0.0834 (3)	0.35158 (15)	0.0286 (7)	
H1A	−0.0132	−0.0853	0.3911	0.034*	
C2	−0.0768 (3)	−0.1405 (3)	0.31582 (17)	0.0345 (8)	
H2A	−0.1351	−0.1809	0.3317	0.041*	
C3	−0.0631 (3)	−0.1360 (3)	0.25741 (17)	0.0326 (8)	
H3A	−0.1124	−0.1727	0.2332	0.039*	
C4	0.0263 (3)	−0.0752 (3)	0.23440 (16)	0.0277 (7)	
C5	0.0465 (3)	−0.0641 (3)	0.17420 (17)	0.0368 (9)	
H5A	−0.0009	−0.0988	0.1483	0.044*	
C6	0.1321 (3)	−0.0049 (4)	0.15423 (15)	0.0384 (9)	
H6A	0.1427	0.0009	0.1147	0.046*	
C7	0.2082 (3)	0.0498 (3)	0.19250 (16)	0.0288 (8)	
C8	0.3005 (3)	0.1110 (3)	0.17423 (17)	0.0342 (8)	
H8A	0.3138	0.1208	0.1352	0.041*	
C9	0.3705 (3)	0.1557 (3)	0.21347 (16)	0.0349 (8)	
H9A	0.4322	0.1958	0.2015	0.042*	
C10	0.3491 (3)	0.1410 (3)	0.27237 (16)	0.0307 (8)	
H10A	0.3973	0.1726	0.2989	0.037*	
C11	0.1920 (3)	0.0382 (3)	0.25240 (15)	0.0231 (7)	
C12	0.0994 (3)	−0.0230 (3)	0.27382 (14)	0.0225 (6)	
C13	0.3092 (2)	0.2624 (3)	0.43099 (13)	0.0187 (6)	
C14	0.3641 (3)	0.3299 (3)	0.47056 (14)	0.0219 (6)	
C15	0.4553 (3)	0.2838 (3)	0.49769 (16)	0.0327 (8)	
H15A	0.4938	0.3266	0.5247	0.039*	
C16	0.4885 (3)	0.1736 (3)	0.48438 (18)	0.0379 (9)	
H16A	0.5515	0.1428	0.5008	0.045*	
C17	0.4264 (3)	0.1104 (3)	0.44629 (16)	0.0306 (8)	
H17A	0.4467	0.0353	0.4386	0.037*	
C18	0.3260 (3)	0.4482 (3)	0.48823 (15)	0.0268 (7)	
C19	0.2113 (2)	0.3025 (3)	0.39622 (14)	0.0184 (6)	
O1W	0.2841 (4)	0.6974 (4)	0.57381 (19)	0.0825 (14)	
H1WA	0.2747	0.6382	0.5532	0.124*	
H1WB	0.2737	0.7562	0.5531	0.124*	

### Atomic displacement parameters (Å^2^)

**Table:**

	*U*^11^	*U*^22^	*U*^33^	*U*^12^	*U*^13^	*U*^23^
Zn1	0.0332 (3)	0.0191 (3)	0.0316 (3)	−0.00121 (15)	−0.00367 (16)	−0.00071 (14)
O1	0.0252 (12)	0.0133 (11)	0.0364 (13)	−0.0015 (9)	−0.0122 (10)	−0.0010 (9)
O2	0.0259 (12)	0.0095 (11)	0.0390 (13)	0.0011 (9)	−0.0060 (10)	−0.0012 (9)
O3	0.089 (3)	0.049 (2)	0.0371 (18)	0.0081 (17)	0.0260 (17)	−0.0036 (14)
O4	0.0411 (14)	0.0166 (11)	0.0286 (12)	−0.0053 (10)	−0.0077 (10)	−0.0003 (9)
N1	0.0236 (14)	0.0206 (13)	0.0247 (13)	−0.0018 (11)	−0.0032 (11)	0.0009 (11)
N2	0.0279 (14)	0.0189 (13)	0.0257 (14)	−0.0032 (11)	−0.0027 (11)	0.0006 (11)
N3	0.0252 (14)	0.0138 (12)	0.0260 (13)	−0.0005 (10)	−0.0049 (11)	0.0020 (10)
C1	0.0278 (17)	0.0292 (18)	0.0288 (18)	−0.0025 (14)	−0.0008 (14)	0.0031 (14)
C2	0.0253 (17)	0.0304 (19)	0.048 (2)	−0.0060 (14)	−0.0013 (16)	0.0016 (16)
C3	0.0268 (17)	0.0309 (19)	0.040 (2)	−0.0039 (15)	−0.0074 (15)	−0.0068 (15)
C4	0.0266 (17)	0.0252 (17)	0.0314 (18)	0.0019 (13)	−0.0067 (14)	−0.0047 (13)
C5	0.037 (2)	0.046 (2)	0.0278 (18)	0.0025 (17)	−0.0112 (16)	−0.0066 (15)
C6	0.043 (2)	0.051 (2)	0.0213 (17)	0.0007 (18)	−0.0068 (15)	−0.0019 (16)
C7	0.0320 (19)	0.031 (2)	0.0229 (17)	0.0047 (14)	−0.0040 (13)	0.0016 (13)
C8	0.043 (2)	0.033 (2)	0.0273 (18)	0.0008 (16)	0.0061 (15)	0.0068 (15)
C9	0.039 (2)	0.0272 (18)	0.039 (2)	−0.0074 (15)	0.0070 (16)	0.0053 (15)
C10	0.0335 (18)	0.0228 (17)	0.0358 (19)	−0.0083 (14)	−0.0003 (15)	0.0011 (14)
C11	0.0262 (16)	0.0192 (15)	0.0238 (16)	0.0014 (12)	−0.0047 (13)	0.0005 (12)
C12	0.0229 (16)	0.0193 (15)	0.0253 (16)	0.0003 (12)	−0.0051 (12)	0.0002 (12)
C13	0.0217 (14)	0.0157 (14)	0.0188 (14)	−0.0006 (11)	−0.0015 (11)	0.0016 (11)
C14	0.0279 (16)	0.0152 (14)	0.0227 (15)	−0.0050 (12)	−0.0033 (12)	0.0014 (11)
C15	0.036 (2)	0.0255 (18)	0.0363 (19)	−0.0051 (14)	−0.0167 (16)	0.0013 (14)
C16	0.034 (2)	0.0265 (19)	0.053 (2)	0.0010 (15)	−0.0228 (18)	0.0048 (16)
C17	0.0296 (18)	0.0182 (16)	0.044 (2)	0.0040 (13)	−0.0109 (15)	0.0016 (14)
C18	0.0351 (19)	0.0217 (17)	0.0237 (16)	−0.0009 (13)	−0.0046 (14)	−0.0049 (12)
C19	0.0218 (15)	0.0156 (14)	0.0178 (14)	0.0004 (11)	−0.0007 (11)	−0.0003 (11)
O1W	0.121 (4)	0.069 (3)	0.057 (3)	0.019 (2)	−0.026 (2)	−0.006 (2)

### Geometric parameters (Å, °)

**Table:**

Zn1—O1	2.102 (2)	C5—C6	1.336 (6)
Zn1—O2^i^	2.110 (2)	C5—H5A	0.9300
Zn1—O4^i^	2.062 (2)	C6—C7	1.436 (5)
Zn1—N1	2.142 (3)	C6—H6A	0.9300
Zn1—N2	2.139 (3)	C7—C8	1.401 (5)
Zn1—N3	2.131 (3)	C7—C11	1.413 (5)
O1—C19	1.262 (4)	C8—C9	1.355 (6)
O2—C19	1.252 (4)	C8—H8A	0.9300
O3—C18	1.221 (5)	C9—C10	1.405 (5)
O4—C18	1.265 (4)	C9—H9A	0.9300
N1—C1	1.324 (4)	C10—H10A	0.9300
N1—C12	1.365 (4)	C11—C12	1.429 (5)
N2—C10	1.331 (5)	C13—C14	1.386 (4)
N2—C11	1.362 (4)	C13—C19	1.518 (4)
N3—C17	1.333 (4)	C14—C15	1.389 (5)
N3—C13	1.349 (4)	C14—C18	1.516 (4)
C1—C2	1.407 (5)	C15—C16	1.386 (5)
C1—H1A	0.9300	C15—H15A	0.9300
C2—C3	1.369 (6)	C16—C17	1.381 (5)
C2—H2A	0.9300	C16—H16A	0.9300
C3—C4	1.408 (5)	C17—H17A	0.9300
C3—H3A	0.9300	O1W—H1WA	0.85
C4—C12	1.417 (5)	O1W—H1WB	0.85
C4—C5	1.427 (5)		
			
O4^i^—Zn1—O1	91.81 (9)	C5—C6—H6A	119.3
O4^i^—Zn1—O2^i^	89.56 (9)	C7—C6—H6A	119.3
O1—Zn1—O2^i^	167.49 (9)	C8—C7—C11	117.4 (3)
O4^i^—Zn1—N3	88.28 (10)	C8—C7—C6	124.0 (4)
O1—Zn1—N3	78.00 (9)	C11—C7—C6	118.6 (3)
O2^i^—Zn1—N3	89.61 (10)	C9—C8—C7	120.0 (3)
O4^i^—Zn1—N2	172.42 (11)	C9—C8—H8A	120.0
O1—Zn1—N2	86.07 (10)	C7—C8—H8A	120.0
O2^i^—Zn1—N2	94.09 (11)	C8—C9—C10	119.4 (3)
N3—Zn1—N2	98.36 (11)	C8—C9—H9A	120.3
O4^i^—Zn1—N1	95.17 (11)	C10—C9—H9A	120.3
O1—Zn1—N1	98.93 (10)	N2—C10—C9	122.6 (3)
O2^i^—Zn1—N1	93.33 (10)	N2—C10—H10A	118.7
N3—Zn1—N1	175.47 (10)	C9—C10—H10A	118.7
N2—Zn1—N1	78.01 (11)	N2—C11—C7	122.4 (3)
C19—O1—Zn1	115.6 (2)	N2—C11—C12	117.5 (3)
C19—O2—Zn1^ii^	138.9 (2)	C7—C11—C12	120.1 (3)
C18—O4—Zn1^ii^	118.5 (2)	N1—C12—C4	122.9 (3)
C1—N1—C12	117.6 (3)	N1—C12—C11	117.8 (3)
C1—N1—Zn1	129.3 (2)	C4—C12—C11	119.3 (3)
C12—N1—Zn1	113.1 (2)	N3—C13—C14	122.4 (3)
C10—N2—C11	118.1 (3)	N3—C13—C19	113.6 (3)
C10—N2—Zn1	128.3 (2)	C14—C13—C19	124.0 (3)
C11—N2—Zn1	113.5 (2)	C13—C14—C15	117.9 (3)
C17—N3—C13	118.8 (3)	C13—C14—C18	123.5 (3)
C17—N3—Zn1	126.6 (2)	C15—C14—C18	118.5 (3)
C13—N3—Zn1	112.7 (2)	C16—C15—C14	119.7 (3)
N1—C1—C2	123.5 (3)	C16—C15—H15A	120.2
N1—C1—H1A	118.3	C14—C15—H15A	120.2
C2—C1—H1A	118.3	C17—C16—C15	118.7 (3)
C3—C2—C1	119.2 (3)	C17—C16—H16A	120.7
C3—C2—H2A	120.4	C15—C16—H16A	120.7
C1—C2—H2A	120.4	N3—C17—C16	122.4 (3)
C2—C3—C4	119.4 (3)	N3—C17—H17A	118.8
C2—C3—H3A	120.3	C16—C17—H17A	118.8
C4—C3—H3A	120.3	O3—C18—O4	126.0 (3)
C3—C4—C12	117.4 (3)	O3—C18—C14	116.0 (3)
C3—C4—C5	123.6 (3)	O4—C18—C14	117.9 (3)
C12—C4—C5	119.1 (3)	O2—C19—O1	123.7 (3)
C6—C5—C4	121.6 (3)	O2—C19—C13	119.2 (3)
C6—C5—H5A	119.2	O1—C19—C13	117.1 (3)
C4—C5—H5A	119.2	H1WA—O1W—H1WB	108.7
C5—C6—C7	121.4 (3)		
			
O4^i^—Zn1—O1—C19	88.5 (2)	Zn1—N2—C11—C7	177.2 (3)
O2^i^—Zn1—O1—C19	−7.6 (6)	C10—N2—C11—C12	−179.9 (3)
N3—Zn1—O1—C19	0.6 (2)	Zn1—N2—C11—C12	−2.9 (4)
N2—Zn1—O1—C19	−98.8 (2)	C8—C7—C11—N2	−0.2 (5)
N1—Zn1—O1—C19	−176.0 (2)	C6—C7—C11—N2	177.4 (3)
O4^i^—Zn1—N1—C1	−4.8 (3)	C8—C7—C11—C12	179.9 (3)
O1—Zn1—N1—C1	−97.5 (3)	C6—C7—C11—C12	−2.4 (5)
O2^i^—Zn1—N1—C1	85.0 (3)	C1—N1—C12—C4	0.6 (5)
N2—Zn1—N1—C1	178.5 (3)	Zn1—N1—C12—C4	179.1 (3)
O4^i^—Zn1—N1—C12	176.9 (2)	C1—N1—C12—C11	179.6 (3)
O1—Zn1—N1—C12	84.2 (2)	Zn1—N1—C12—C11	−1.9 (4)
O2^i^—Zn1—N1—C12	−93.3 (2)	C3—C4—C12—N1	−1.8 (5)
N2—Zn1—N1—C12	0.2 (2)	C5—C4—C12—N1	178.4 (3)
O1—Zn1—N2—C10	78.1 (3)	C3—C4—C12—C11	179.2 (3)
O2^i^—Zn1—N2—C10	−89.4 (3)	C5—C4—C12—C11	−0.7 (5)
N3—Zn1—N2—C10	0.8 (3)	N2—C11—C12—N1	3.3 (4)
N1—Zn1—N2—C10	178.1 (3)	C7—C11—C12—N1	−176.9 (3)
O1—Zn1—N2—C11	−98.5 (2)	N2—C11—C12—C4	−177.7 (3)
O2^i^—Zn1—N2—C11	94.0 (2)	C7—C11—C12—C4	2.2 (5)
N3—Zn1—N2—C11	−175.8 (2)	C17—N3—C13—C14	−3.6 (5)
N1—Zn1—N2—C11	1.5 (2)	Zn1—N3—C13—C14	161.9 (2)
O4^i^—Zn1—N3—C17	81.9 (3)	C17—N3—C13—C19	176.9 (3)
O1—Zn1—N3—C17	174.1 (3)	Zn1—N3—C13—C19	−17.7 (3)
O2^i^—Zn1—N3—C17	−7.7 (3)	N3—C13—C14—C15	3.1 (5)
N2—Zn1—N3—C17	−101.8 (3)	C19—C13—C14—C15	−177.4 (3)
O4^i^—Zn1—N3—C13	−82.2 (2)	N3—C13—C14—C18	−173.2 (3)
O1—Zn1—N3—C13	10.0 (2)	C19—C13—C14—C18	6.3 (5)
O2^i^—Zn1—N3—C13	−171.8 (2)	C13—C14—C15—C16	0.4 (5)
N2—Zn1—N3—C13	94.2 (2)	C18—C14—C15—C16	176.8 (4)
C12—N1—C1—C2	1.3 (5)	C14—C15—C16—C17	−3.1 (6)
Zn1—N1—C1—C2	−176.9 (3)	C13—N3—C17—C16	0.6 (5)
N1—C1—C2—C3	−1.9 (6)	Zn1—N3—C17—C16	−162.6 (3)
C1—C2—C3—C4	0.5 (6)	C15—C16—C17—N3	2.7 (6)
C2—C3—C4—C12	1.2 (5)	Zn1^ii^—O4—C18—O3	−109.1 (4)
C2—C3—C4—C5	−179.0 (4)	Zn1^ii^—O4—C18—C14	74.6 (4)
C3—C4—C5—C6	179.6 (4)	C13—C14—C18—O3	95.4 (5)
C12—C4—C5—C6	−0.6 (6)	C15—C14—C18—O3	−80.8 (5)
C4—C5—C6—C7	0.3 (6)	C13—C14—C18—O4	−87.9 (4)
C5—C6—C7—C8	178.7 (4)	C15—C14—C18—O4	95.8 (4)
C5—C6—C7—C11	1.2 (6)	Zn1^ii^—O2—C19—O1	−143.6 (3)
C11—C7—C8—C9	0.4 (5)	Zn1^ii^—O2—C19—C13	35.2 (5)
C6—C7—C8—C9	−177.1 (4)	Zn1—O1—C19—O2	168.5 (3)
C7—C8—C9—C10	−0.5 (6)	Zn1—O1—C19—C13	−10.3 (4)
C11—N2—C10—C9	−0.4 (5)	N3—C13—C19—O2	−159.6 (3)
Zn1—N2—C10—C9	−176.9 (3)	C14—C13—C19—O2	20.9 (5)
C8—C9—C10—N2	0.5 (6)	N3—C13—C19—O1	19.2 (4)
C10—N2—C11—C7	0.3 (5)	C14—C13—C19—O1	−160.3 (3)

Symmetry codes: (i) −*x*+1/2, *y*−1/2, *z*; (ii) −*x*+1/2, *y*+1/2, *z*.

### Hydrogen-bond geometry (Å, °)

**Table:**

*D*—H···*A*	*D*—H	H···*A*	*D*···*A*	*D*—H···*A*
O1W—H1WA···O3	0.85	2.22	3.022 (5)	157
O1W—H1WB···O3^ii^	0.85	2.42	3.215 (5)	157

Symmetry codes: (ii) −*x*+1/2, *y*+1/2, *z*.
